# CDK9 and SPT5 proteins are specifically required for expression of herpes simplex virus 1 replication-dependent late genes

**DOI:** 10.1074/jbc.M117.806000

**Published:** 2017-07-25

**Authors:** Zhiyuan Zhao, Ka-Wei Tang, Isabella Muylaert, Tore Samuelsson, Per Elias

**Affiliations:** From the Institute of Biomedicine, Department of Medical Biochemistry and Cell Biology, Sahlgrenska Academy, University of Gothenburg, Box 440, SE-405 30 Gothenburg, Sweden

**Keywords:** cyclin-dependent kinase (CDK), gene expression, herpesvirus, post-transcriptional regulation, viral replication

## Abstract

DNA replication greatly enhances expression of the herpes simplex virus 1 (HSV-1) γ2 late genes by still unknown mechanisms. Here, we demonstrate that 5,6-dichloro-1-β-d-ribofuranosylbenzimidazole (DRB), an inhibitor of CDK9, suppresses expression of γ2 late genes with an IC_50_ of 5 μm, which is at least 10 times lower than the IC_50_ value required for inhibition of expression of early genes. The effect of DRB could not be explained by inhibition of DNA replication *per se* or loading of RNA polymerase II to late promoters and subsequent reduction of transcription. Instead, DRB reduces accumulation of γ2 late mRNA in the cytoplasm. In addition, we show that siRNA-mediated knockdown of the transcription factor SPT5, but not NELF-E, also gives rise to a specific inhibition of HSV-1 late gene expression. Finally, addition of DRB reduces co-immunoprecipitation of ICP27 using an anti-SPT5 antibody. Our results suggest that efficient expression of replication-dependent γ2 late genes is, at least in part, regulated by CDK9 dependent co- and/or post-transcriptional events involving SPT5 and ICP27.

## Introduction

Viruses are under strong selection to produce new infectious particles in cells. To achieve this goal, viruses coordinate synthesis of new genomes with synthesis of capsid proteins and assembly of the virion. The infectious cycle of herpes simplex virus 1 (HSV-1) illustrates this phenomenon. The expression of γ2 late genes, encoding *e.g.* capsid proteins and glycoproteins, is either strictly dependent on or strongly stimulated by viral DNA synthesis (1–3). The molecular mechanisms responsible for coordinating DNA replication and gene expression are poorly understood.

The molecular mechanisms underlying the tightly regulated program for gene expression executed during lytic infection by HSV-1 have been extensively studied for several decades. In the classical version three classes of genes are expressed in a coordinated fashion (1, 2) during the HSV-1 infectious life cycle. First, a complex of the viral VP16, OCT1, and other host cell transcription factor acts on TAATGARAT elements upstream of promoters for immediate early genes (α genes) (4). Second, transcription of early genes (β genes) encoding enzymes required for DNA synthesis is activated by the immediate early gene product ICP4. Finally, the late genes (γ genes), which also require ICP4, are turned on when DNA replication starts (5). Some late genes, the γ1 genes, are not completely dependent on DNA synthesis, whereas the expression of γ2 genes is drastically reduced by suppression of HSV-1 DNA synthesis. An influential report has demonstrated that trans-acting factors present before or during replication are not enough to support γ2 gene expression indicating the existence of cis-acting mechanisms tightly associated with viral DNA synthesis (3).

Detailed analyses of early and late promoters suggest that upstream sequences govern expression of early genes and that downstream regulatory elements are important for expression of late genes (6–12). The importance of downstream regulatory elements in late gene expression is further demonstrated by an observation that RNA polymerase II can be loaded on HSV-1 late promoters in the presence of a DNA synthesis inhibitor without supporting gene expression, which suggests additional regulatory mechanisms acting downstream of promoter recognition (13). Such mechanisms might include the positive transcription elongation factor b (P-TEFb)^3^ and involve release of transcription complexes from promoter-proximal stalling as well as control of elongation checkpoints further downstream of gene promoter and close to polyadenylation signals (14, 15). Indeed, it was found that the drug 5,6-dichloro-1-β-d-ribofuranosylbenzimidazole (DRB), which is an inhibitor of the CDK9 activity of P-TEFb, repressed expression of some but not all late gene expression (16–19). Further evidence of CDK9 involvement in HSV-1 gene expression was supported by the observation that the transcription factor SPT5, which together with SPT4 is a part of DRB-sensitive inducing factor (DSIF), was localized to HSV-1 DNA near the viral replication fork (20, 21).

P-TEFb is composed of CDK9 and a regulatory subunit cyclin T (14). CDK9 is a kinase known to regulate several processes coupled to transcription (14, 15, 22, 23). Three important substrates for CDK9 are RNA polymerase II, NELF-E, and DSIF (14, 22–28). In cells, DSIF becomes associated with RNA polymerase II after initiation of transcription and, together with the negative elongation factor NELF, acts to stall transcription in a promoter proximal position (14, 24). The stalled transcription complexes are released after the phosphorylation of SPT5 and NELF by CDK9, and phosphorylated SPT5 turns into an elongation factor (25–28).

Here, we have embarked on a series of experiments aimed at identifying factors essential for expression of HSV-1 DNA replication-dependent genes. To start, by using ChIP technology we examined the kinetics and genetic requirements of RNA polymerase II binding to immediate early, early, and late promoters. In agreement with previous observations, we found that loading of RNA polymerase II on the late UL38 promoter requires the ICP4 transcription factor but was independent of DNA replication (13). This observation suggests that co- and post-transcriptional mechanisms play a significant role in regulating HSV-1 late gene expression. We also observed that DRB selectively affected the synthesis of γ2 late gene products with a much lower IC_50_ value than early gene expression. A direct role of CDK9 on γ2 late gene expression was then demonstrated by an experiment in which addition of DRB 7 h post-infection caused a drastic and specific inhibition of synthesis of HSV-1 late proteins. We also found that knockdown of SPT5 led to a profound reduction of synthesis of the late gene product glycoprotein C. In contrast, knockdown of NELF-E did not specifically affect late gene expression. Finally, we observed that ICP27 could be immunoprecipitated by an antibody directed against SPT5 and that this interaction was affected by DRB. We speculate that this finding may, at least partially, explain the reduced accumulation of late mRNA in the cytoplasm of infected cells induced by DRB and less efficient translation of viral mRNAs (29).

## Results

### Requirements for loading of RNA polymerase II on early and late HSV-1 promoters

**Figure 1.**
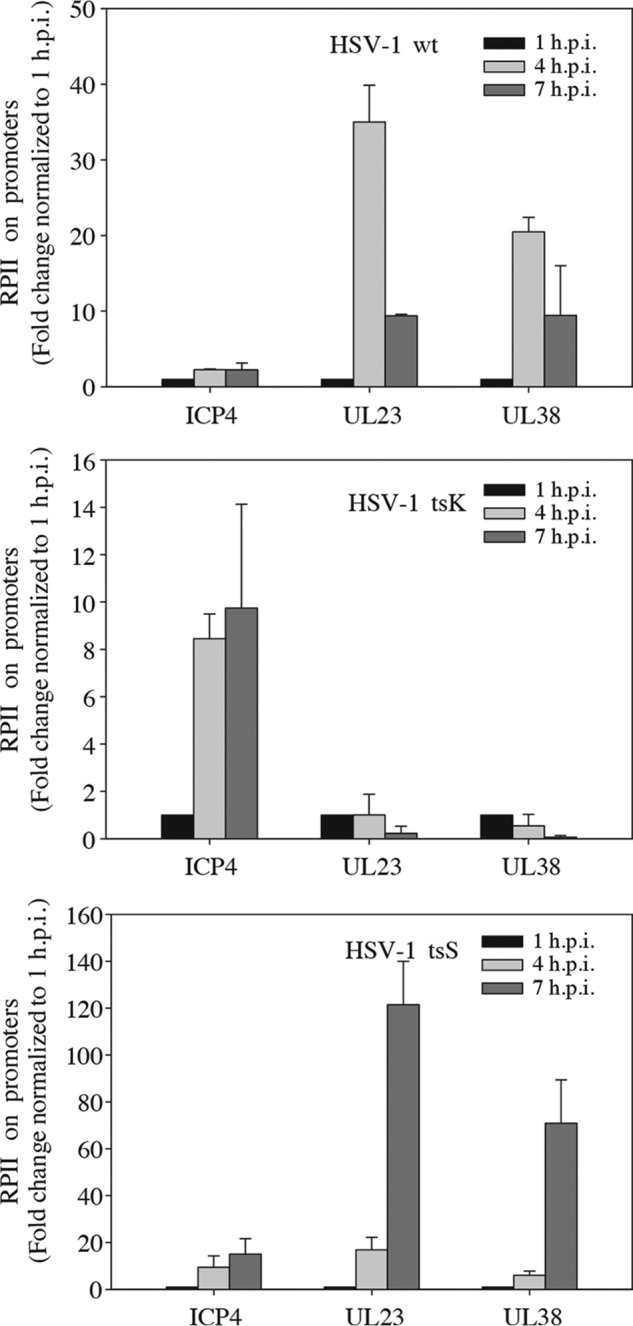
**Recruitment of RNA polymerase II to HSV-1 promoters.** ChIP against RNA polymerase II (*RPII*) was performed in HSV-1-infected 1BR.3.N cells using either WT virus or mutant virus tsK defective in ICP4 or tsS defective in the UL9 protein. The cells were infected at the non-permissive temperature using an m.o.i. of 7 at 0 h post-infection (*h.p.i.*). They were harvested at the indicated time points. The values obtained for each ChIP experiment were normalized to the value obtained for the 1 h.p.i. The mean value of two independent experiments is displayed, and the *error bars* indicate variation.

To establish a starting point, we reinvestigated the kinetics as well as the genetic requirements for loading of RNA polymerase II on the promoters controlling expression of the immediate early transcriptional regulator ICP4, the early gene encoding thymidine kinase UL23 and the late gene UL38 encoding a capsid protein. Regulation dependent on cis-acting elements for these genes has previously been examined in some detail (9, 30–32). We made use of two temperature-sensitive mutants: first, tsK, affecting the transcription factor ICP4, and second, tsS, affecting the UL9 origin-binding protein, required for initiation of DNA synthesis (33, 34). We examined binding of RNA polymerase II to viral promoters using ChIP analyses. The experiments were performed at the non-permissive temperatures for the temperature-sensitive mutants, 39 °C, at an m.o.i. of 7. The results were normalized to the value obtained for binding of RNA polymerase II to immediate early, early, and late promoters, respectively, at 1 h after addition of virus to the medium. In the text and in the figures this time point is defined as 1 hour post-infection (h.p.i.) (Fig. 1).

For wild-type virus the results showed efficient loading of RNA polymerase II on immediate early ICP4, early UL23, and late UL38 promoters (Fig. 1, *upper panel*). We also noted that the occupancy of RNA polymerase II on immediate early promoters changed little over time. In contrast, binding of RNA polymerase II to the UL23 and UL38 promoters increased substantially at 4 h.p.i. At 7 h.p.i., when DNA synthesis had started, the promoter occupancy began to decrease.

Infection with the tsK mutant revealed binding of RNA polymerase II to the immediate early promoter but not to a significant extent to the early and the late promoters (Fig. 1, *middle panel*). In contrast, infection with the tsS mutant led to a pronounced accumulation of RNA polymerase II at both early and late promoters at 4 and 7 h.p.i. (Fig. 1, *lower panel*).

The results show, in agreement with a previous study, that ICP4 and not DNA replication is required to recruit RNA polymerase II to both early and late promoters (13). Furthermore, the decrease in promoter occupancy for the wild-type virus at 7 h.p.i. indicated that DNA replication and the increasing amounts of viral DNA led to reduced average occupancy of RNA polymerase II on virus DNA.

### Effects of DRB on RNA polymerase II distribution on an early and late HSV-1 gene

**Figure 2.**
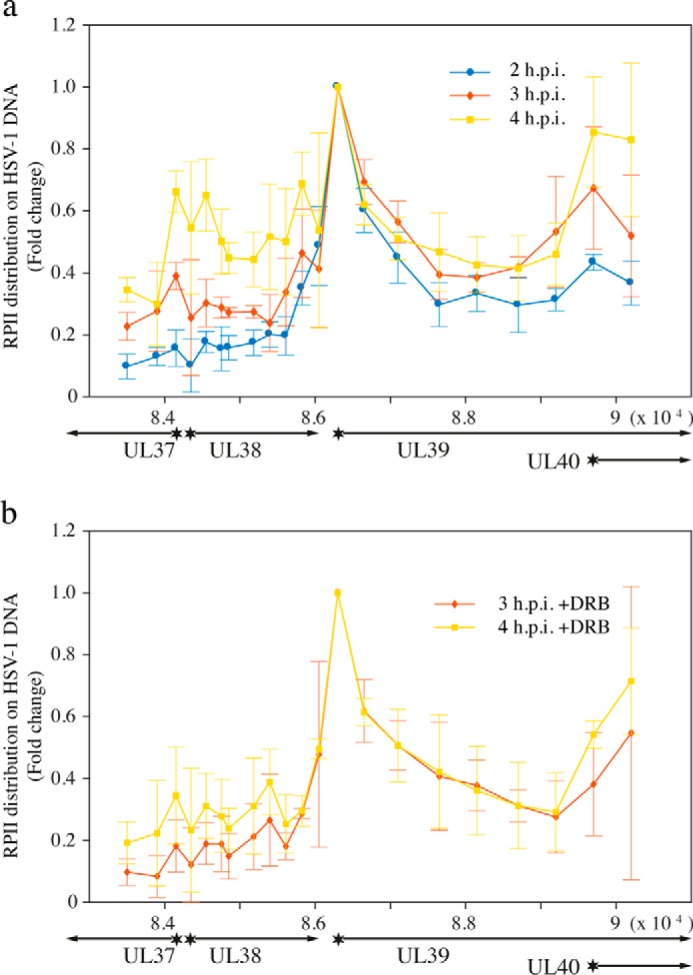
**Effect of DRB on the distribution of RNA polymerase II on an early (UL39) and a late (UL38) gene.** ChIP against RNA polymerase II was performed in 1BR.3.N cells infected with HSV-1 at 37 °C using an m.o.i. of 10. Virus was added to cells at 0 h, and cells were harvested at the indicated time points. Three independent experiments were performed, and for each experiment the individual observations were normalized using the UL39 promoter as reference. The mean value for each experimental point was calculated, and the *error bars* show the standard deviation. Experiments performed in the absence of DRB (*a*) and in the presence of 25 μm DRB (*b*).

On the basis of the results presented above, we reasoned that differential regulation of early and late gene expression might involve control mechanisms operating downstream of loading of RNA polymerase II on promoters. CDK9 is a well-known regulator of gene transcription downstream of promoter loading (14, 15, 22–28). Previous studies have shown that DRB, a specific inhibitor of CDK9, reduces the expression of the UL38 gene (19). A pilot study was therefore performed to characterize the distribution of RNA polymerase II over two neighboring genes, the late gene UL38 and the early gene UL39, at 2, 4, and 7 h.p.i. using ChIP-qPCR (supplemental Fig. 1). Guided by our results, we then made a more detailed study looking at the effects of DRB on RNA polymerase II distribution on these genes. Cells were infected with HSV-1, in the presence and absence of 25 μm DRB, at an m.o.i. of 10 and harvested at 2–4 h.p.i. (Fig. 2). In the absence of DRB, RNA polymerase II already found its way to the early UL39 gene 2 h.p.i., but only later, at 3 h.p.i., it bound efficiently to the UL38 gene (Fig. 2*a*). At 4 h.p.i. the distribution of RNA polymerase II on the UL38 and UL39 genes was similar. In the presence of DRB, we found that the series of events observed in the absence of the drug was delayed by about 1 h (Fig. 2*b*). In fact, the overall signal to noise ratio for RNA polymerase II distribution at 2 h.p.i. in the presence of DRB was too low at some positions to allow reliable calculations. At 3 and 4 h.p.i., the pattern observed was similar to the patterns observed at 2 and 3 h.p.i. in the absence of DRB.

**Figure 3.**
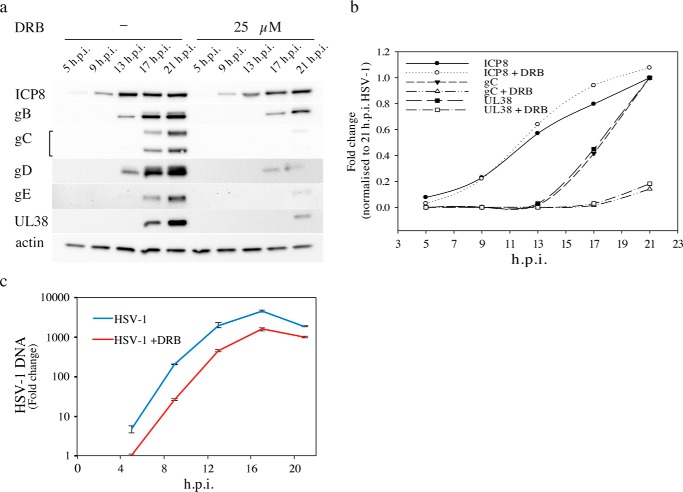
**Inhibition of late but not early gene expression by DRB is accompanied by a delay of HSV-1 DNA replication.** *a,* time course of HSV-1 gene expression at 37 °C with an m.o.i. of 1 was examined with immunoblot. Experiments were performed in the presence or absence of 25 μm DRB. *b,* gene expression was quantified in the immunoblot. The mean value of two independent experiments is displayed. *c,* HSV-1 DNA replication was measured by qPCR in three independent experiments. The *error bars* show the standard deviation.

The effect of DRB on HSV-1 DNA synthesis was addressed with qPCR (supplemental Fig. 2). It shows that the amount of virus DNA increased 10-fold between 4 and 7 h.p.i., and that DRB caused a delay in the onset of DNA replication. However, once started, the rate of DNA synthesis was unaffected by DRB (Fig. 3*c*).

### Expression of late but not early genes is inhibited by DRB

The combined effects of DRB on the expression of some early and late genes was examined in a time-course experiment. A previous study reported that DRB reduced expression of the UL38 gene but not the UL44 gene encoding glycoprotein C (19). These genes were therefore included in this study. Here, the experiments were performed at 37 °C using an m.o.i. of 1 to avoid over-riding possible regulatory events. The kinetics of expression of an early protein ICP8 (the single-strand DNA-binding protein) and the late proteins gB, gC, gD, gE, and UL38 were examined in the absence or presence of 25 μm DRB (Fig. 3, *a* and *b*). We noted that the time course for accumulation of ICP8 was only slightly altered by the addition of DRB. In contrast, we found that expression of the γ2 genes, gC, gD, gE, and UL38, was strongly inhibited by addition of the drug. Expression of gB, which has previously been found to have intermediate characteristics (35, 36), was not as dramatically affected by DRB.

**Figure 4.**
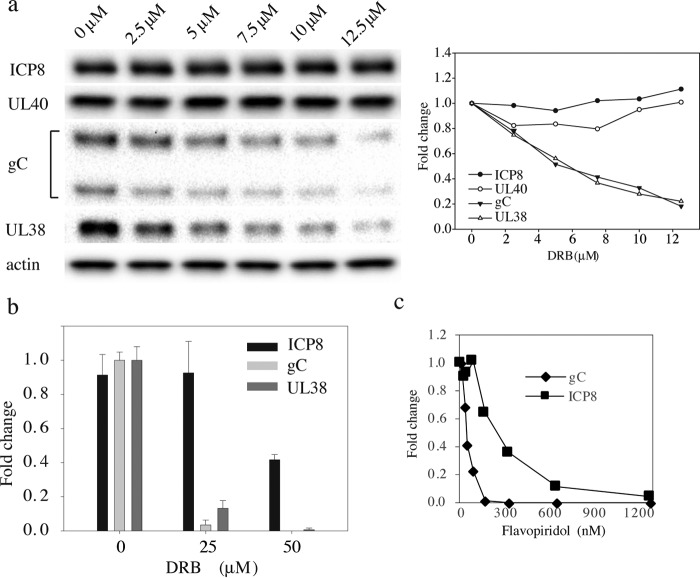
**Determination of the IC_50_ for DRB and flavopiridol for early and late gene expression.** HSV-1 gene expression was measured at 19 h.p.i. at an m.o.i. of 1 by immunoblotting in the presence of DRB (*a* and *b*) or flavopiridol (*c*) at the concentrations indicated. *a* and *c,* quantification shows the mean value of two experiments and is normalized to the mock-treated infections. *b,* quantification of protein expression from three independent experiments, and *error bars* indicate standard deviation.

To make sure we were observing a specific effect on late gene expression, we determined the apparent IC_50_ value for inhibition of gene expression by DRB (Fig. 4*a*). We found that expression from the late genes, UL38 and gC, were efficiently inhibited with an estimated IC_50_ of 5 μm. Significant effects on early gene expression, ICP8, was only seen at concentrations approaching 50 μm (Fig. 4*b*). We also looked at the effects of DRB at an m.o.i. of 10, to facilitate a comparison with the ChIP analyses (supplemental Fig. 3). Here, we found an IC_50_ for gC of 7 μm, and the IC_50_ was more than 80 μm for ICP8. Importantly, the IC_50_ value observed for late gene expression is close to the estimated value for inhibiting HIV transcription (37). It is therefore likely to reflect a specific inhibition of CDK9. We also examined effects of flavopiridol, a CDK9 inhibitor used in clinical trials, on HSV-1 gene expression and observed a specific inhibition of late gene expression with an IC_50_ of 40 nm (Fig. 4*c*). The IC_50_ for ICP8 was >200 nm.

### Knockdown of SPT5 inhibits late gene expression

**Figure 5.**
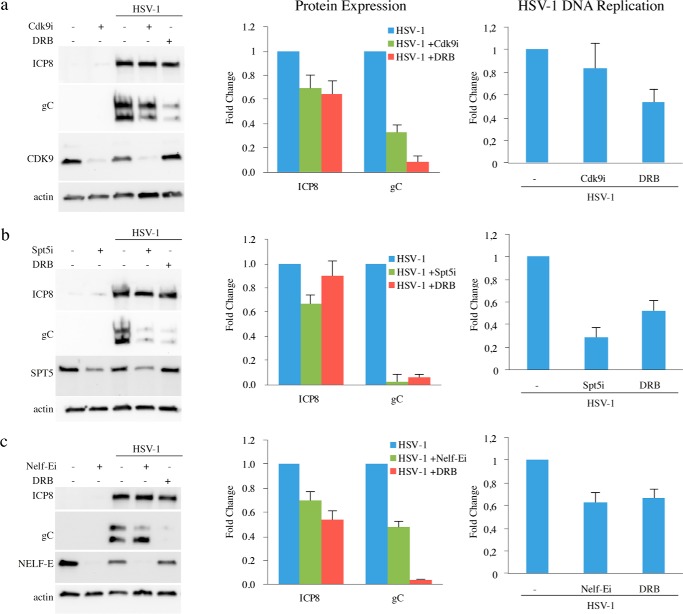
**Effects of siRNA-mediated knockdown of CDK9, SPT5, and NELF-E on HSV-1 gene expression and DNA replication.** siRNAs for CDK9 (*a*), SPT5 (*b*), and NELF-E (*c*) were used in knockdown experiments in 1BR.3.N cells. After the knockdown, the cells were infected with HSV-1 and harvested 19 h.p.i. for either immunoblot or qPCR. As comparison, cells with no knockdown were infected with HSV-1 in the absence and presence of DRB in parallel to the knockdown experiments. Each experiment was subjected to three independent repeats, and the mean value is displayed in the diagrams. *Error bars* indicate standard deviation.

The molecular mechanism underlying the inhibitory effect of DRB on late gene expression was analyzed in siRNA knockdown experiments directed toward CDK9, SPT5, and NELF-E. The efficiency of knockdown and HSV-1 gene expression was assessed by Western blotting (Fig. 5). The residual amounts of CDK9, SPT5, and NELF-E obtained in three independent experiments varied between 2 and 30%.

Knockdown of CDK9 reduced the expression of gC by 70%. A more modest reduction of 30% for the expression of ICP8 was observed. At the same time, HSV-l DNA synthesis was largely unaffected (Fig. 5*a*). Knockdown of CDK9 by siRNA primarily affects the inactive 7SK small nuclear RNP complexes, and the addition of DRB is therefore likely to cause a more complete inhibition of the kinase activity of CDK9 (38). Consequently, less gC was observed in the presence of DRB.

We then observed that knockdown of SPT5 by siRNA had a severe effect on expression of gC, reducing its expression by more than 90% (Fig. 5*b*). Again, only a 30% reduction of ICP8 expression was seen (Fig. 5*b*). In addition, we also observed the amount of HSV-1 DNA was reduced by 70%. Finally, knockdown of NELF-E reduced expression of ICP8 by 30% and gC by 50%. HSV-1 DNA synthesis was reduced by 50% (Fig. 5*c*).

To summarize, in the siRNA knockdown effect we saw a modest reduction of the expression of ICP8 by 30% in all instances. In stark contrast, knockdown of SPT5 caused a reduction of gC expression by more than 90%. Our results therefore suggest that SPT5 plays a crucial role in the viral life cycle in particular for supporting expression of γ2 late genes. In contrast, our results fail to reveal a specific role for NELF-E in regulating early and late gene expression.

### Inhibitory effect of DRB on HSV-1 late gene expression cannot solely be explained by altered transcription

**Figure 6.**
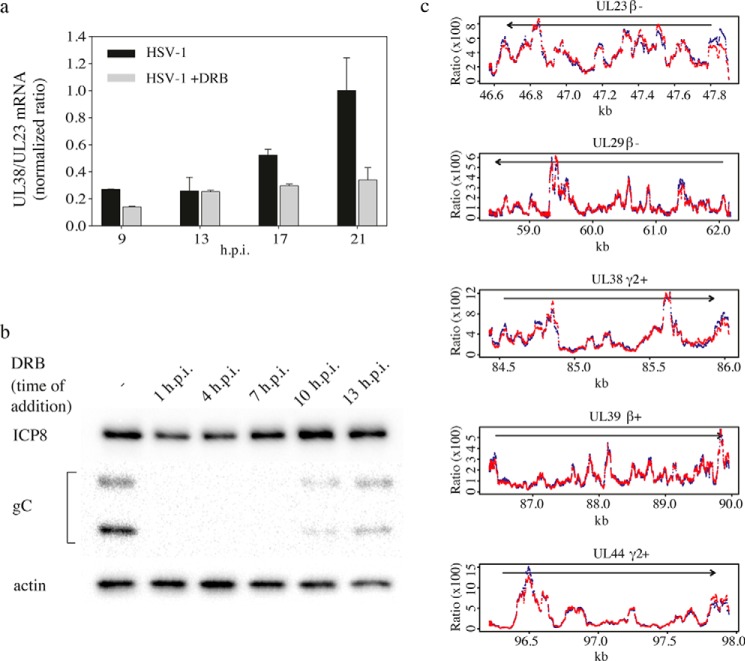
**Effects of DRB on HSV-1 early and late transcripts, and DRB can still exert full inhibitory effect on a late gene expression added 7 h.p.i.** *a,* ratios between UL38 and UL23 mRNAs were measured by multiplex RT-qPCR in cells infected with HSV-1 at an m.o.i. of 1. Then 25 μm DRB was added 1 h.p.i. in parallel with undisturbed infection. Cells were harvested at the indicated time. The mean values of three independent experiments are displayed, and the *error bars* indicate the standard deviation. *b,* expression of ICP8, glycoprotein C, and actin was measured by Western blotting at 37 °C at 19 h.p.i. at an m.o.i. of 1. The time of addition of 25 μm DRB to the culture medium is shown. *c,* HSV-1 transcriptome in cells infected with HSV-1 at an m.o.i. of 1 was analyzed at 13 h.p.i. by high throughput RNA sequencing. Sequencing was performed on infected cells in the absence of DRB (*red*) and in cells supplied with 25 μm DRB at 7 h.p.i. (*blue*). The results are expressed as ratio between reads mapping to a specific position relative to the total number of reads mapping to that gene.

The prominent inhibition of late but not early gene expression could conceivably be caused by direct effects on transcription or RNA stability. We first examined, using RT-qPCR, the ratio between mRNAs from a late, UL38, and an early, UL23, gene during the progression of an HSV-1 infection (Fig. 6*a*). In the absence of DRB this ratio increased ∼3-fold, from 0.3 to 1, between 9 and 21 h.p.i. In the presence of DRB, the ratio changed from 0.1 to 0.3, suggesting that DRB suppressed the accumulation of late mRNA. However, the effect was not of a magnitude that would easily explain the effects of DRB on early and late protein levels.

To pinpoint where in the viral life cycle DRB exerts its most direct effect on late gene expression, we performed a time of addition experiment (Fig. 6*b*). In this experiment, we added 25 μm DRB at different times after an infection with HSV-1 at an m.o.i. of 1 and harvested cells at 19 h.p.i. (Fig. 6*b*). Protein expression was then analyzed using Western blotting. We found that DRB could be added as late as 7 h.p.i. and still exert full inhibitory effect on late gene expression, but no effect on synthesis of ICP8 was observed. At this time, DNA replication was well on its way (Fig. 3*c* and supplemental Fig. 2). Also, DRB significantly inhibited late gene expression when added as late as at 13 h.p.i. The results suggest that DRB prevents late gene expression during ongoing viral DNA synthesis and that CDK9 is continuously required throughout the infection.

To get a global picture of the effects of DRB on HSV-1 gene expression, we looked at the HSV-1 transcriptome. Guided by the time of addition experiment, we added DRB 7 h.p.i. to separate effects of the drug on immediate early and early gene transcription from those on late gene transcription. Cells were harvested at 13 h.p.i. The result showed that the number of reads mapping to the HSV-1 genome was reduced 2-fold, from 11 to 6.9%, in the presence of DRB (Table 1). At the individual gene level, reads mapped to the viral genes were normalized to the total number of reads mapped to HSV-1, and the ratios, in the presence or absence of DRB, were calculated (supplemental Table 2). A ratio above 1 indicated that the transcript was relatively enriched in DRB-treated cells. The values ranged between 1.69 for UL23, thymidine kinase, and 0.64 for UL44, glycoprotein C. Eleven of 12 early genes had values higher than 1, and conversely, late transcripts were slightly more abundant in the untreated cells. When the number of reads at a specific position of a gene were normalized to the total number of reads mapped to that specific gene for early genes, UL23, UL29, and UL39, and to late genes, UL38 and UL44, the patterns were close to identical in the presence and absence of DRB (Fig. 6*c*). The results suggest that, under these conditions, 25 μm DRB exerted only a small effect on global virus transcription.

**Table 1:** **Effect of DRB on HSV-1 transcription** RNA-sequencing experiments are shown.

	Total reads	Reads mapped to HSV-1	Percent reads mapped to HSV-1
Mock	8,855,884	274	0.0031%
HSV-1*^a^*	3,826,721	425,319	11%
HSV-1 + DRB*^b^*	3,725,564	257,961	6.9%

*^a^* The 1BR.3.N monolayers were infected with HSV-1 at an m.o.i. of 1. Cells were harvested 13 h post-infection.

*^b^* The 1BR.3.N monolayers were infected with HSV-1 at an m.o.i. of 1. Then 25 μm DRB was added 6 h post-infection, and cells were harvested 13 h post-infection.

### Inhibition of P-TEFb by DRB prevents accumulation of late mRNA in the cytoplasm of infected cells

The results described in the previous paragraphs suggest that post-transcriptional events instigated by CDK9 may play a significant role in explaining how DRB affects viral gene expression. Here, one should consider mRNA maturation, transport, and subsequent translation.

**Figure 7.**
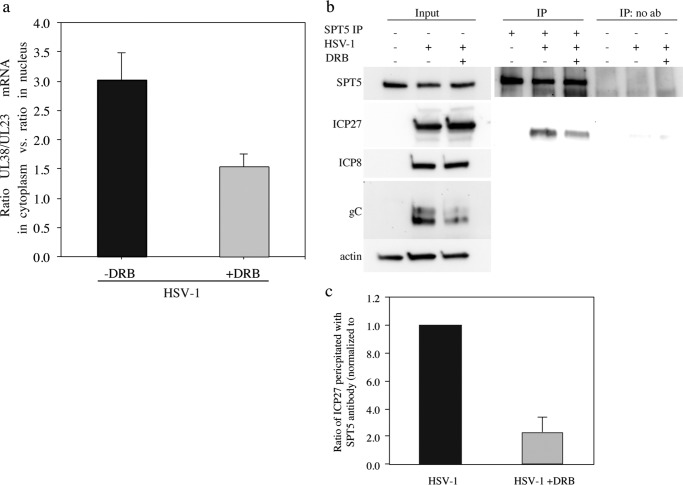
**DRB reduces cytoplasmic accumulation of HSV-1 mRNA and the amount of ICP27 co-immunoprecipitated with SPT5.** *a,* 1BR.3.N cells were infected with HSV-1 at an m.o.i. of 1. RNA was isolated from nuclear and cytoplasmic fractions obtained by subcellular fractionation. The ratio between UL38 and UL23 mRNA in the cytoplasm and the nucleus was determined by multiplex RT-qPCR at 19 h.p.i. in four independent experiments. The results are displayed as the mean value of the quotient between cytoplasmic and nuclear ratios in the presence (*black*) or absence of 25 μm DRB (*gray*), and the *error bars* indicate standard deviation. The statistical significance determined by unpaired two-tailed Student's *t* test with unequal variance was *p* = 0.0074. *b,* immunoblot from an anti-SPT5 immunoprecipitation experiment using extracts from HSV-1-infected HeLa cells. The cells were infected at an m.o.i. of 10 and treated either with 25 μm DRB or with DMSO at 1 h.p.i. At 17 h.p.i., the cells were harvested and submitted to anti-SPT5 immunoprecipitations. *c,* quantification of three independent immunoprecipitation experiments. The ratios between the measured amounts of ICP27 and SPT5 from experiments performed in the presence or absence of DRB were calculated. The ratios obtained in the absence of DRB were used for normalization. The *error bars* indicate standard deviation.

We first investigated whether DRB affected the accumulation of late γ2 mRNA in the cytoplasm. An experiment was performed in which cells infected with HSV-1 in the presence or absence of 25 μm DRB were subjected to subcellular fractionation. The nuclear and cytosolic fractions were then used to quantify the ratio of UL38/UL23 mRNA by RT-qPCR. The results were displayed as a quotient of the ratio of UL38/UL23 mRNA in the cytoplasm divided by the ratio of UL38/UL23 mRNA in the nucleus. Our results demonstrate that this quotient was reduced by 50% in the presence of DRB (Fig. 7*a*). The difference in accumulation of γ2 mRNA in the cytosol observed in the presence of DRB supports the notion that inhibition of CDK9 may affect maturation, transport, and translation of late mRNA.

### DRB prevents the association between ICP27 and SPT5

Several reports demonstrate an involvement of the essential HSV-1 immediate early gene product ICP27 in co- and post-transcriptional processes as well as possible direct effects on translation (12, 29, 39–43).

We reasoned that one mechanism for CDK9 to promote expression of γ2 late genes would be to facilitate a possible SPT5-dependent loading of ICP27 to mRNA. An interaction between the C-terminal domain of RNA polymerase II and ICP27 has previously been described. However, this interaction appears to be insensitive to phosphorylation (44). We therefore performed co-immunoprecipitation experiments in extracts from HSV-1-infected HeLa cells, grown in the presence or absence of DRB, using an antibody against SPT5. We readily detected an interaction between ICP27 and SPT5 in the absence of DRB (Fig. 7*b*). Intriguingly, addition of DRB reduced the amount of ICP27 in the co-immunoprecipitate by 5-fold (Fig. 7*c*).

In summary, our results support the notion that ICP27 associates directly or indirectly with SPT5 most probably bound to RNA polymerase II in a CDK9-dependent manner to enhance nuclear export and possibly translation of γ2 late mRNAs.

## Discussion

This study was initiated to search for molecular mechanisms underlying coupling of HSV-1 DNA replication to gene expression. We initially confirmed that loading of RNA polymerase II to promoters occurred independently of DNA replication (13). We then explored the possibility that co-transcriptional and post-transcriptional mechanism might help to explain the well-established observation that γ2 late genes depend on on-going DNA replication for efficient expression. CDK9 in complex with cyclin T makes up the positive elongation factor b, P-TEFb, and it is known to exert prominent effects on transcriptional regulation downstream of loading of RNA polymerase II to promoters (14).

Most commonly, CDK9 is associated with release of RNA polymerase II from promoter proximal stalling by phosphorylating, in addition to serine 2 residues in the heptapeptide repeat of RNA polymerase II, SPT5 and NELF-E. The activity of CDK9 is regulated at several levels (18, 45). It is predominantly kept in an inactive form with 7SK RNA and HEXIM1/2, and the active form is recruited to support gene expression by different mechanisms (45). A classical example concerns regulation of HIV transcription. In this instance, the HIV Tat protein is capable of forming a complex with P-TEFb to act on stalled transcription complexes at the HIV TAR sequence (46, 47).

DRB has been shown to effectively inhibit the activity of CDK9 (16, 39). The selectivities of DRB and flavopiridol in relation to other CDKs have been extensively examined demonstrating at least a 5–10-fold difference in favor of CDK9 *versus* other CDKs such as CDK1, -2, -4, -6, and -7 (16–18). We have now used DRB in a quantitative manner to explore the role of CDK9 in cells infected with HSV-1. As expected, we observed that DRB affected several aspects of the progression of an HSV-1 infection. During the early stages of the infection, we found that loading of RNA polymerase II on late genes was delayed, a phenomenon that might be related to delayed expression of immediate early genes (48). We also noted that the onset of DNA synthesis was delayed. However, once DNA synthesis had started, the rate of DNA replication was unaffected.

To explore the effect of DRB on gene expression, we measured the amounts of a selected set of early and late genes by Western blotting. We found that DRB efficiently inhibited synthesis of γ2 late genes with an IC_50_ of 5 μm, which is similar to the effect of DRB on HIV gene expression (37). In contrast, DRB had little or no effect on the expression of early genes such as UL29 and UL40. Significant inhibition of early gene expression occurred only at concentrations of DRB above 50 μm.

We next sought to explore whether SPT5 and NELF-E, direct targets of CDK9 phosphorylation, might play a role for regulating HSV-1 gene expression. The recent observation that SPT5 is associated with HSV-1 DNA and is close to the viral replication fork (20, 21) strengthens this hypothesis. In addition to take part in promoter proximal stalling and transcription elongation, SPT5 may also recruit components involved in mRNA maturation (14, 49–52). We performed siRNA knockdown experiments directed against CDK9, SPT5, and NELF-E. The most striking results was a more than 10-fold suppression of gC expression when SPT5 was knocked down. Intriguingly, only a minor 30% reduction of expression of the UL29 gene encoding ICP8 was observed. DNA replication was reduced to 30% as compared with untreated cells. Our observation suggests an essential role for SPT5 in regulating late gene expression. In contrast, knocking down NELF-E, the negative elongation factor, had no clear selective effect on late gene expression, indicating that it does not exert a major influence on selective expression of late *versus* early genes. Finally, in knockdown experiments directed against CDK9, we observed selective reduction of the late UL44 gene encoding gC *versus* the early gene UL29 encoding ICP8. DRB, however, was more effective in reducing gC expression. In this case we favor an explanation based on the observation that siRNA preferentially causes a reduction of the CDK9 in its inactive complex and thus a comparatively smaller reduction of CDK9 kinase activity (38).

Because CDK9 and SPT5 are required for late gene expression, we sought to explore possible direct effects on transcription and accumulation of mRNA. First, we measured by RT-qPCR the ratios of a late, UL38, mRNA to an early, UL23, mRNA and observed that DRB caused a reduced accumulation of the late mRNA. The effect could reflect a general delay in the progress of the infection. Therefore, we performed an experiment in which DRB was added at different times after infection. Interestingly, we found that DRB could be added as late as 7 h.p.i. and still exert full inhibitory effect on late gene expression. Prominent inhibition was also seen when DRB was added as late as 10 and 13 h.p.i. Using this information we performed an experiment in which we added DRB at 7 h.p.i. and measured mRNA levels by RNA sequencing at 13 h.p.i. We noted only a 2-fold decrease in the total amount of viral RNA synthesis (Table 1). The reduction was more prominent for late mRNAs than for early mRNAs (supplemental Table S2).

However, the magnitude of inhibition does not explain the more drastic effect observed for protein expression. We then proceeded to look at the post-transcriptional fate of viral mRNA by measuring mRNA levels in nuclear and cytoplasmic compartments. Interestingly, we noted in the presence of DRB a relative decrease in the amount of late mRNA reaching the cytoplasm. It suggested to us that CDK9 might regulate a co-transcriptional process promoting maturation and transport of late mRNAs. To further explore this possibility, we looked for a DRB-sensitive interaction between SPT5 and the viral protein ICP27, which is known to promote nuclear transport and subsequent translation (29, 39–43). We measured the ratios between ICP27 and SPT5 in immunoprecipitates brought down by an antibody against SPT5 and found a 5-fold decrease of ICP27 in extracts from cells treated with DRB.

The results presented here reveal that inhibition of CDK9 by DRB has multiple effects on HSV-1 infection. Notably, it delays progression of the infection by interfering with loading of RNA polymerase II primarily on late genes and a subsequent delay in the start of viral DNA synthesis. Interestingly, it also has a specific effect on late gene expression most likely involving co-transcriptional events resulting in CDK9, and SPT5 promoted recruitment of ICP27 to transcriptional complexes. These still poorly characterized steps appear to result in a slightly lower production of late mRNAs accompanied by impaired nuclear export. It remains to be explained why these steps preferentially affect late gene expression. Possibly profound alterations of the cellular environment reflected in global disruption of transcription need to be counteracted at later times (53, 54). It is also possible that there exists a specific mechanism by which CDK9 is recruited to replicating DNA to promote replication-dependent gene expression. The latter alternative might directly involve viral and cellular proteins taking part in DNA synthesis, but no evidence for such a mechanism is at hand. A specific role for CDK9 in regulating late gene expression is likely to involve structural elements in late genes or the corresponding transcript. At least for one late gene, UL44 encoding gC, there is evidence for sequences strongly affecting gene expression (12). It would be of interest to see whether such regulatory elements respond to CDK9 as well as ICP27.

## Experimental procedures

### Cells and viruses

1BR.3.N human fibroblast cells and HeLa cells were grown in Dulbecco's modified Eagle's medium supplemented with 10% fetal calf serum. The viruses used were as follows: HSV-1 (WT), strain 17 syn+, and the temperature-sensitive mutant viruses tsK, an A475V mutant defective in ICP4 (30), and tsS, an A90T mutant defective in UL9 protein (31), both derived from strain 17 syn+. Virus was propagated in BHK-21 cells, and the experiments were performed using either 1BR.3.N or HeLa cells.

### Antibodies

Anti-RNA polymerase II (ab5408) used for ChIP, anti-β-actin (ab6276), anti-CDK9 (ab76320), anti-NELF-E (ab170104), anti-SPT5 (ab26259 and ab89219), anti-ICP8 (20194), and ICP27 (ab53480) used for Western blotting antibodies were purchased from Abcam. The anti-SPT5 (sc-28678x) antibody is directed to an epitope corresponding to amino acids 61–360 mapping near the N terminus of SPT5 of human origin and was used for co-immunoprecipitation. The anti-ICP27 (sc-17544) antibody was used for Western blotting. Both antibodies were purchased from Santa Cruz Biotechnology. Additional antibodies used for Western blotting were as follows: a mouse monoclonal anti-UL40 antibody was kindly provided by Lars Thelander (Umeå University), a rabbit polyclonal anti-ICP8 was provided by Maria Falkenberg (University of Gothenburg), and mouse monoclonal anti-glycoprotein B, anti-glycoprotein C, anti-glycoprotein D, and anti-glycoprotein G antibodies were generously provided by Tomas Bergström (University of Gothenburg).

### Chemicals

5,6-Dichloro-1-β-d-ribofuranosylbenzimidazole (DRB), catalogue no. D1916, was purchased from Sigma and dissolved in absolute ethanol or in DMSO. Flavopiridol (catalogue no. A10390) was purchased from AdooQ BioScience (Irvine, CA) and was dissolved in 70% ethanol.

### Cell infection

1BR.3.N cells were grown in 6-well plates, 24-well plates, T75 flasks, or 500-cm^2^ dishes as indicated, and HeLa cells were grown in P100 dishes. Cells were infected with 1, 7, or 10 plaque-forming units of virus per cell in a medium consisting of Dulbecco's modified Eagle's medium supplemented with 2% fetal calf serum. After 1 h of incubation at 37 or 39 °C, the inoculum was replaced with growth medium alone and supplemented with DRB and flavopiridol as indicated. Controls were treated identically with the appropriate amount of solvent. The cultures were then incubated at 37 or 39 °C until harvested.

### Chromatin immunoprecipitation

ChIP was performed according to Affymetrix Chromatin Immunoprecipitation Assay Protocol with minor changes. Briefly, 1BR.3N cells were grown on 500-cm^2^ plates or T75 flasks and infected as described above. Cells were cross-linked using 1% formaldehyde, quenched with 125 mm glycine, and washed twice with ice-cold phosphate-buffered saline (PBS). The cells were collected using a rubber policeman and pelleted by low speed centrifugation. The cells were lysed in lysis buffer, according to the protocol, and nuclei were washed three times using the same buffer. The nuclear pellet was resuspended in pre-IP dilution buffer with protease inhibitor (Roche Applied Science) and with 0.8% SDS and sonicated to obtain chromatin fragments of about 250 bp (Bioruptor UCD-200; Diagenode). Fragmented chromatin was then diluted in IP dilution buffer and pre-cleared using protein G-Sepharose beads (GE Healthcare). Anti-polymerase II (ab5408) was added to each tube and incubated overnight at 4 °C. Immunoprecipitation was carried out with protein G-Sepharose beads and collected in an Ultrafree-MC tube (Millipore). Beads were washed with ChIP Wash 1, ChIP Wash 2, and ChIP Wash 3 before being eluted with Elution Buffer. Incubation of beads at 65 °C overnight reversed the cross-link, and DNA was extracted with phenol extraction and precipitated using ethanol precipitation. Enriched ChIP-DNA was quantified using qPCR (see below).

### Immunoblotting

Following infection, cells were lysed and subjected to Western blot analyses as described previously (55). The expression levels were quantified using Chemidox XRS.

### siRNA transfection

Duplex siRNA for Spt5 (5′-AACTGGGCGAGTATTACATGA-3′) (56) was ordered from Eurofins MWG Operon (Ebersberg, Germany), and pre-designed siRNA for CDK9 (s2834) and for NELF-E (138780) were bought from Thermo Fisher Scientific. 1BR.3.N monolayers grown to 40% confluency on 24-well collagen-coated plates were transfected with 25 pmol of siRNA by using Oligofectamine (Invitrogen) according to the supplier's manual. Three days after transfection, the cells were infected with HSV-1 at an m.o.i. of 1 and harvested at 19 h.p.i. for immunoblotting and DNA extraction. Controls were treated in an identical manner but without siRNA.

### DNA extraction

1BR.3.N cells were grown on 6- or 24-well plates following siRNA treatment and infected as described above. Cells were washed with PBS, lysed using Buffer AL (QIAamp DNA Blood Mini kit, Qiagen), and homogenized using Qiashredder (Qiagen). Subsequent steps for DNA extraction were performed according to the supplier's instructions.

### RNA extractions

1BR.3.N cells were grown in 6-well plates or T75 flasks and infected as described above. Cells were washed with PBS and lysed using Denaturation buffer ToTALLY RNA^TM^ kit (Ambion) and homogenized using Qiashredder (Qiagen). Subsequent steps for RNA extraction were performed according to the supplier's instructions. Samples were subjected to rigorous DNase treatment using TURBO DNA-free kit (Ambion) and RNA purification (RNeasy Mini Kit, Qiagen). For transcriptome sequencing (RNA-Seq), samples were further processed using Ribo-Zero rRNA removal kit (Epicenter) and analyzed using Experion RNA analysis kit (Bio-Rad). Library preparation and sequencing on Illumina HiSeq 2000 were carried out by BGI (Shenzhen, China).

### Subcellular fractionation

RNA isolation following subcellular fractionation was performed using 1BR.3.N cells grown and infected as described above. The cells were harvested using brief trypsin treatment and collected by centrifugation. They were then resuspended in Dulbecco's modified Eagle's medium and transferred to Falcon tubes. The cells were spun down at 550 × *g* for 5 min at 4 °C and washed with ice-cold PBS. The cells were then lysed in a buffer containing 10 mm Tris-HCl, pH 8, 140 mm NaCl, 1.5 mm MgCl_2_, 0.5% Nonidet P-40 substitute, 1000 units/ml RNase inhibitor, and 1× Protease inhibitor. The soluble cytoplasmic fraction was collected after centrifugation, 550 × *g* for 5 min at 4 °C. The pellet containing the nuclear fraction was washed with buffer containing 51.5 mm Tris-HCl, pH 8, 134.5 mm NaCl, 27.4 mm MgCl_2_, 0.6% Tween 20, 0.3% sodium deoxycholate, 1000 units/ml RNase inhibitor, and 1× protease inhibitor. The RNA from the cytoplasmic and nuclear fractions was extracted with TRIzol according to the manufacturer's protocol (Life Technologies, Inc.). RNA was DNase-treated and purified as described above.

### RT-qPCR and qPCR

cDNA from purified RNA samples was created by using iScript cDNA synthesis kit (Bio-Rad) according to the manufacturer's instructions. Purified DNA, ChIP-DNA, and cDNA was quantified by using iQ SYBR Green Supermix (Bio-Rad) or iQ Multiplex Powermix (Bio-Rad). Primer sequences are provided in supplemental Table 1.

### Bioinformatics

The total number of reads from each RNA-Seq experiment, see above, as well as reads aligned to the HSV-1 genome is shown in supplemental Table 2. Reads were aligned to a sequence database containing both human (genome assembly GRCh37) and HSV-1 (GenBank^TM^ accession number X14112.1) genomes. Alignment was performed using bwa (57). Read coverage was calculated using coverageBed of the bedtools suite (58) and current annotation of HSV-1 genes. Data were processed and plotted using in-house Perl scripts and R. Complete sequence information is available at NCBI Sequence Read archive accession number SRP034653.

### Co-immunoprecipitation

HeLa cells were grown in monolayer in P100 dishes to 90% confluency and infected with HSV-1 at an m.o.i. of 10. At 17 h.p.i., the cells were lysed with M-PER mammalian protein extraction reagent (Thermo Fisher Scientific) supplemented with NaCl (to final concentration of 0.15 m), EDTA (to final concentration 0.002 m), Complete protease inhibitors mix, and PhosStop (Roche Applied Science). The cell lysates were cleared by centrifugation at 14,000 × *g* for 15 min followed by an overnight incubation with an antibody against SPT5 (sc-28678x) at 4 °C. Subsequently, Pierce protein A/G magnetic beads (Thermo Fisher Scientific) were added to the lysates, and immunoprecipitations were performed as recommended by the manufacturer. The solubilized immunoprecipitates were subjected to electrophoresis and Western blotting as described earlier.

## Author contributions

Z. Z., K. W. T., and P. E. designed the study and wrote the paper. K. W. T. performed the ChIP experiments. Z. Z. and K. W. T. examined protein expression, RT-qPCR experiments, and DNA synthesis. Z. Z. performed the subcellular fractionation and the siRNA experiments. Z. Z. and I. M. performed the co-immunoprecipitation experiments. T. S. performed the bioinformatics analysis. All authors analyzed the results and approved the final version of the manuscript.

## Supplementary Material

Supplemental Data

## Footnotes

3 The abbreviations used are:

P-TEFb: positive transcription elongation factor b
DSIF: DRB sensitivity-inducing factor
DRB: 5,6-dichloro-1-β-d-ribofuranosylbenzimidazole
m.o.i.: multiplicity of infection
h.p.i.: hours postinfection
g: glycoprotein
qPCR: quantitative PCR
CDK: cyclin-dependent kinase
RNA-Seq: RNA-sequencing.
