# Supplementary material for: CDK9 and SPT5 proteins are specifically required for expression of herpes simplex virus 1 replication-dependent late genes
Source: J Biol Chem. 2017 Jul 25;292(37):15489–500. doi: 10.1074/jbc.M117.806000 (PMC5602406; doi:10.1074/jbc.M117.806000)
Supplement: Supplemental Data [file supp_292_37_15489__index.html]

Cdk9 and Spt5 are specifically required for expression of Herpes simplex virus 1 replication-dependent late genes. — CDK9 and SPT5 proteins are specifically required for expression of herpes simplex virus 1 replication-dependent late genes — Control of HSV-1 late gene expression — Supplemental Data 

# CDK9 and SPT5 proteins are specifically required for expression of herpes simplex virus 1 replication-dependent late genes

## Supplemental Data

- Supplementary figure 1 (.pdf, 368 KB) - Distrubition of RPII on UL38 and UL39 genes
- Supplementary figure 2 (.pdf, 362 KB) - Effects of DRB on HSV-1 replication
- Supplementary figure 3 (.pdf, 416 KB) - Determination of IC50 for inhibiton by DRB of HSV-1 gene expression at m.o.i. 10
- Supplementary table 1 (.pdf, 34 KB) - Primers for qPCR and RT-qPCR
- Supplementary table 2 (.pdf, 73 KB) - Effects of DRB on the HSV-1 transcriptome
