# Supplementary material for: CDK9 and SPT5 proteins are specifically required for expression of herpes simplex virus 1 replication-dependent late genes
Source: J Biol Chem. 2017 Jul 25;292(37):15489–500. doi: 10.1074/jbc.M117.806000 (PMC5602406; doi:10.1074/jbc.M117.806000)
Supplement: Supplemental Data [file 10.1074_M117.806000_jbc.M117.806000-1.pdf]

Figure S1

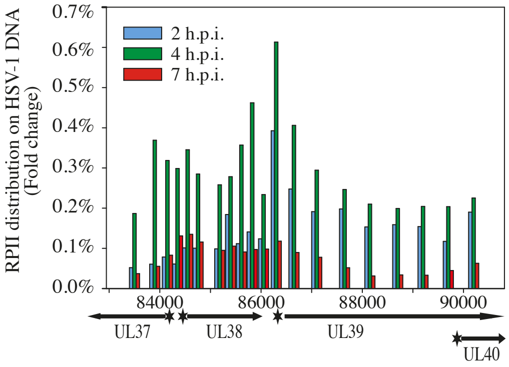

ChIP against RNA polymerase II was performed in 1BR.3.N cells infected with HSV-1 in at 37°C using a m.o.i. of 10. Virus was added to cells at 0 hour and cells were harvested at 2 (blue), 4 (green) and 7 (red) h.p.i.. The values show specific enrichment of RNA polymerase II bound to DNA as percentage of input DNA.
