# Supplementary material for: CDK9 and SPT5 proteins are specifically required for expression of herpes simplex virus 1 replication-dependent late genes
Source: J Biol Chem. 2017 Jul 25;292(37):15489–500. doi: 10.1074/jbc.M117.806000 (PMC5602406; doi:10.1074/jbc.M117.806000)
Supplement: Supplemental Data [file 10.1074_M117.806000_jbc.M117.806000-2.pdf]

Figure S2

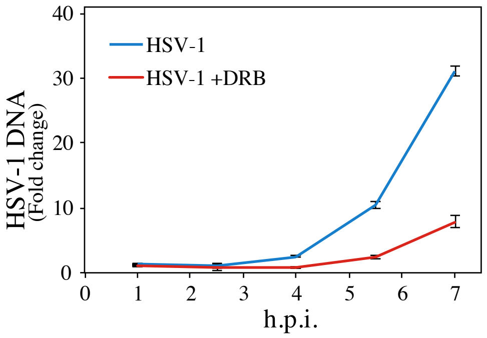

HSV-1 DNA replication was measured by quantitative PCR in the presence of 25 μM DRB or DMSO. The results were normalized to the 1 h.p.i. and displayed as fold change. The mean value of two independent experiments is displayed and error bars indicate the
