# Supplementary material for: CDK9 and SPT5 proteins are specifically required for expression of herpes simplex virus 1 replication-dependent late genes
Source: J Biol Chem. 2017 Jul 25;292(37):15489–500. doi: 10.1074/jbc.M117.806000 (PMC5602406; doi:10.1074/jbc.M117.806000)
Supplement: Supplemental Data [file 10.1074_M117.806000_jbc.M117.806000-3.pdf]

Figure S3

a

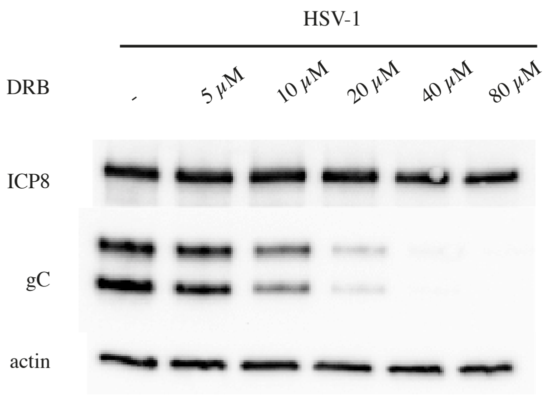

b

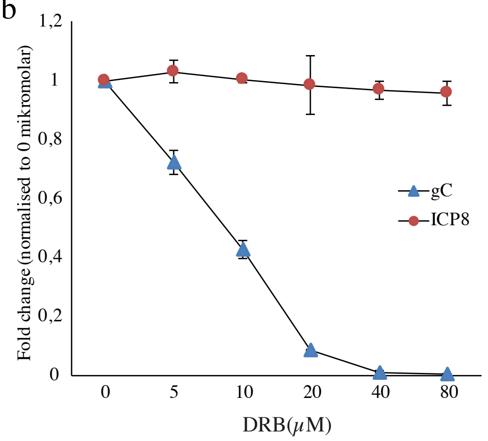

(**a**) HSV-1 gene expression was measured at 19 h.p.i. at a m.o.i. of 10 by immunoblotting in the presence of the indicated concentration of DRB or DMSO. (**b)** Quantification shows the mean value of two independent experiments and is normalized to the mock treated infections. Error bars indicate the variation.
