# Supplementary material for: CDK9 and SPT5 proteins are specifically required for expression of herpes simplex virus 1 replication-dependent late genes
Source: J Biol Chem. 2017 Jul 25;292(37):15489–500. doi: 10.1074/jbc.M117.806000 (PMC5602406; doi:10.1074/jbc.M117.806000)
Supplement: Supplemental Data [file 10.1074_M117.806000_jbc.M117.806000-4.pdf]

Table S1

Name	Sequence (5'-3')	Position	Product
ChIP 1 (Figure 1)<br>UL38_promoter primer-pair was also used for quantification of DNA (Figure 2B, 6B & 7)			
ICP4_promoter_F<br>ICP4_promoter_R	GGGTCGTGCATAATGGAA<br>CGTCTGACGGTCTGTCTCT	146679<br>146874	195bp
UL23_promoter_F<br>UL23_promoter_R	AGTTTCACGCCACCAAGATCTGC<br>AATTCGAACACGCAGATGCAGTCG	47840<br>47990	150bp
UL38_promoter_F<br>UL38_promoter_R	GTGGGTTGCGGACTTTCTG<br>CTCCGGTGGGTTGTGTTG	84324<br>84418	94bp
RT-qPCR (Figure 8A & 9)			
UL23_middle_F<br>UL23_middle_R	CCCAACACGATGTTTGTGC<br>GGCCCTCACCCTCATCTT	47186<br>47338	152bp
UL23_probe	FAM-ATGCTGCCCATAAGGTATCG-BHQ1	47258	
UL38_middle_F<br>UL38_middle_R	CTGGATCGTTTTTCCGAGTG<br>GCTCGCTAGCTCGTCCTG	85197<br>85313	116bp
UL38_probe	HEX-ATGGTTCACACGCACGTCT-BHQ1	85227	
ChIP 2 (Figure 2A & 3)			
UL37_1_F<br>UL37_1_R	GAGCGACGTCTCCCCAAG<br>CAAACCGTCACCAAGAGCTG	83476<br>83547	71bp
UL37_2_F<br>UL37_2_R	TTCCCGACAACAGAAACG<br>TCGGACGGACCTATGCAG	83868<br>84021	153bp
UL37_3_F<br>UL37_3_R	CGTAGACCAACGACGAGACC<br>GATAATTCGCCGCTCCCTAC	84114<br>84177	63bp
UL38_A_F<br>UL38_A_R	GTGGGTTGCGGACTTTCTG<br>GTTGTGTTGGCCGACTGG	84324<br>84409	85bp
UL38_B_F<br>UL38_B_R	GGTCGCAATGAAGACCAATC<br>GCGGTATCGCGTGTTGTG	84524<br>84613	89bp
UL38_C_F<br>UL38_C_R	CACGCTGTGGTTGCTTGG<br>CCTGTTCGGTATCGTCGTTG	84710<br>84786	76bp
UL38_D_F<br>UL38_D_R	AGGCCCTGGACAAGATCC<br>GTTGGCACAGATCCGTCAG	84784<br>84891	107bp
UL38_E_F<br>UL38_E_R	ACGTCACGGCCAACTACC<br>CACTCGGAAAAACGATCCAG	85156<br>85216	60bp
UL38_F_F<br>UL38_F_R	GCTGGTGTCGTGGGTCAC<br>GCGTCCAGGTCCACGAAC	85277<br>85420	143bp
UL38_G_F<br>UL38_G_R	CCAGGAGCTGTGTTGTGTGT<br>GAAGTCGGGGTTTCGGTTT	85490<br>85646	156bp
UL38_H_F<br>UL38_H_R	CATGATGCCCGAGGATAGTC<br>GCCTTCCAGAATGACAACG	85805<br>85889	84bp
UL38_I_F<br>UL38_I_R	CCGCTGTCACTCGTTGTTC<br>ATTTGCATTTTGTGGCTTCC	85977<br>86088	111bp
UL39_1_F<br>UL39_1_R	CCGTGCTCCTGTGAGCTT<br>AGAGTAGGCGAGAGCAGGTC	86251<br>86326	75bp
UL39_2_F<br>UL39_2_R	AGTCAATGGCGTGATGGTG<br>GGTGCAGTTGGAACCACATT	86587<br>86683	96bp
UL39_3_F<br>UL39_3_R	CTCGGAGACGCTGTCACAC<br>ATGACGAATCGGAGTCAAGG	87058<br>87137	79bp
UL39_4_F<br>UL39_4_R	CCTGTGTCTGGACGTTCCTC<br>CCCAGGATGCGGTAAAGG	87595<br>87726	131bp
UL39_5_F<br>UL39_5_R	CCTCTACGACCACCAGATCG<br>GGTAGCAGCTGGAGGTGTAG	88111<br>88193	82bp
UL39_6_F<br>UL39_6_R	AGATACCCATCCAGGAGCTG<br>GCGTCTTTGAACATGACGAA	88680<br>88761	121bp
UL39_7_F<br>UL39_7_R	CTGTCGGCGATGAAGACC<br>GCGATACATGCTGCGCTTA	89141<br>89221	80bp
UL40_1_F<br>UL40_1_R	TAGCCAATCCATGACCCTGT<br>CTGTTTTTAGTCCGCGCTTA	89662<br>89768	106bp
UL40_2_F<br>UL40_2_R	TCAGCTTTTACCGCTTCCTC<br>CATCGAAAACTACGTGCGATT	90164<br>90268	104bp

Primers for qPCR and ChIP experiments. The position and sequence of the primers are shown. It is also indicated in which experiments the individual primers were used.
