# Supplementary material for: CDK9 and SPT5 proteins are specifically required for expression of herpes simplex virus 1 replication-dependent late genes
Source: J Biol Chem. 2017 Jul 25;292(37):15489–500. doi: 10.1074/jbc.M117.806000 (PMC5602406; doi:10.1074/jbc.M117.806000)
Supplement: Supplemental Data [file 10.1074_M117.806000_jbc.M117.806000-5.pdf]

Table S2

				DRB (-)	DRB (+)	Mock	
start	end	gene	strand	counts	Counts	counts	**DRB+/DRB-** (Normalized to reads mapping to reads mapping to HSV-1)
**46672**	**47802**	**UL23/b**	**-**	**7502**	**7670**	**0**	**1,685695147**
47737	48546	UL24/g1	+	1718	1719	1	1,649732193
113734	115272	UL54/a	+	5447	4902	5	1,483804432
26946	28502	UL13/g	-	873	753	1	1,422137095
28256	28915	UL14/g2	-	411	346	1	1,388017716
**25007**	**26887**	**UL12/b**	**-**	**5822**	**4765**	**2**	**1,349433341**
**18224**	**20476**	**UL8/b**	**-**	**2096**	**1665**	**2**	**1,30973578**
**107010**	**108125**	**UL50/b**	**+**	**3764**	**2946**	**2**	**1,290457958**
127232	131128	RS1/a	-	745	583	2	1,290247464
2261	2317	RL2/a	+	54	42	0	1,282378602
134053	134928	US2/g2	-	1367	1058	0	1,276079951
69633	70460	UL34/g1	+	2509	1902	2	1,249886518
**58463**	**62053**	**UL29/b**	**-**	**7961**	**6034**	**4**	**1,249678834**
24801	25091	UL11/g	-	2552	1899	0	1,226888306
29020	30048	UL15/g	+	464	344	1	1,222365811
48813	50555	UL25/g2	+	2477	1805	0	1,201467236
142744	143223	US8A/	+	1318	957	1	1,197173954
**89926**	**90948**	**UL40/b**	**+**	**9251**	**6632**	**9**	**1,18199753**
**86444**	**89857**	**UL39/b**	**+**	**9981**	**7142**	**7**	**1,179794922**
**20704**	**23259**	**UL9/b**	**-**	**4387**	**3122**	**8**	**1,173345728**
69161	69553	UL33/g2	+	359	253	0	1,1619483
147105	151001	RS1_2/a	+	822	578	1	1,159355838
55802	58159	UL28/g	-	2953	2072	0	1,156876599
141243	142895	US8/g1	+	3859	2660	4	1,136495159
135222	136667	US3/g1	+	2160	1487	0	1,135057727
124054	124110	RL2_6/a	-	77	53	0	1,134869375
67372	69162	UL32/g2	-	1878	129 0	1	1,132543403
**62807**	**66514**	**UL30/b**	**+**	**9663**	**6537**	**5**	**1,115391261**
80713	84084	UL37/g1	-	3897	2632	3	1,113566639
17135	18025	UL7/g1	+	1813	1223	0	1,112216632
66459	67379	UL31/g	-	4293	2862	2	1,099181659
53080	55794	UL27/g1	-	39282	25983	26	1,090577251
112179	113195	UL53/g	+	2217	1449	0	1,077614495
**93113**	**94579**	**UL42/b**	**+**	**10668**	**6934**	**7**	**1,071671207**
51727	52716	UL26.5/g	+	7453	4807	5	1,063417329
11823	12422	UL4/a	-	1240	799	0	1,062394531
50809	52716	UL26/g	+	9504	6114	5	1,060668666
31386	33497	UL17/g	-	1217	773	0	1,047248261
**12483**	**15131**	**UL5/b**	**-**	**2948**	**1870**	**4**	**1,045863146**
143313	143585	US9/g	+	3122	1976	1	1,043553632
115496	116056	UL55/g2	+	1107	696	2	1,036626605
70566	70904	UL35/g2	+	3483	2189	3	1,036222503
43866	46382	UL22/g	-	5785	3621	6	1,032014725
9884	10888	UL2/b/g1	+	3277	2051	2	1,031929318
40820	41488	UL20/g1	-	737	459	1	1,026847452

137731	138009	US5/g	+	333	206	0	1,019961359
30174	31295	UL16/g1	-	1771	1087	0	1,011979501
15130	17160	UL6/g	+	2155	1316	1	1,006860601
94748	96052	UL43/g	+	876	528	0	0,993780678
23204	24625	UL10/g2	+	5311	3171	5	0,984420554
136744	137460	US4/g1	+	5270	3146	0	0,984257732
36404	40528	UL19/g	-	12788	7622	13	0,982713787
84531	85928	UL38/g2	+	2819	1635	1	0,956276346
132644	133906	US1/a	+	13921	8042	8	0,952476715
35095	36051	UL18/g1	-	4373	2519	4	0,949750263
145311	145577	US12/a	-	4233	2436	2	0,948832928
116221	116925	UL56/g2	-	1764	1008	2	0,942155708
33635	34813	UL15_2/g	+	1249	710	0	0,937252576
71049	80543	UL36/g2	-	3148	1775	5	0,929660473
3083	3749	RL2_2/a	+	699	393	2	0,926992258
98796	100952	UL46/g1	-	11267	6232	6	0,911968594
108277	109011	UL51/g1	-	944	521	2	0,909968715
**109048**	**112224**	**UL52/b**	**+**	**3662**	**2005**	**3**	**0,902727701**
105486	106391	UL49/g?	-	10342	5535	5	0,882416914
9337	10011	UL1/g1	+	2884	1500	2	0,857544637
139785	140957	US7/g1	+	9935	5161	9	0,856498723
122622	123288	RL2_5/a	-	718	371	2	0,851942331
138419	139603	US6/g	+	9851	4977	7	0,833005855
98032	98550	UL45/g2	+	4281	2117	1	0,815335519
42074	43681	UL21/g	+	2483	1219	0	0,809445696
103607	105079	UL48/g	-	14375	6975	9	0,800013086
10957	11664	UL3/a	+	1334	629	0	0,777419712
101035	103116	UL47/g1	-	3444	1623	2	0,776991216
144761	145246	US11/g2	-	18454	8494	4	0,758896365
106718	106993	UL49A/	-	888	408	1	0,757544117
144157	145095	US10/g1	-	35080	16080	16	0,755765725
120882	122485	RL2_4/a	-	1263	557	1	0,72713086
3886	5489	RL2_3/a	+	1223	539	1	0,726646256
91168	92637	UL41/g1	-	3106	1361	3	0,722465988
96311	97846	UL44/g2	+	13727	5318	17	0,638753704

Evaluation of RNA-Seq experiments performed in the presence or absence of 25 μM DRB. DRB was added at 6 h.p.i. and cells from the two experiments were harvested at 13 h.p.i.. The total number of reads obtained in the absence of DRB was 3 826 721 and 425 319 reads (11%) mapped to the HSV-1 genome. For the experiment performed in the presence of DRB 3 725 564 reads were obtained and 257 961 reads (6.9%) mapped to the HSV-1 genome. For the mock infection experiment 8 855 884 reads were obtained and 274 mapped to the HSV-1 genome. The early genes (b) are highlighted in bold print. Immediate early and late genes are designated as a, g1 and g2.
